# Supplementary material for: Computational investigation of IP3 diffusion
Source: Sci Rep. 2023 Feb 20;13:2922. doi: 10.1038/s41598-023-29876-3 (PMC9941478; doi:10.1038/s41598-023-29876-3)
Supplement: Supplementary file 1 — Supplementary Information. [file 41598_2023_29876_MOESM1_ESM.docx]

1. **Description of the algorithm**

The algorithm is made of two parts: a deterministic one that uses COMSOL Multiphysics and describes Ca^2+^ diffusion, SERCA pumps and a leak from the ER. The other one is stochastic and is a Gillespie's algorithm developed in MATLAB.

IP_3_R clusters are defined as rectangular regions of 0.5 x 0.5 µm^2^ and their stochastic behaviour was simulated in MATLAB and coupled with the deterministic processes simulated in COMSOL using the Livelink package, which allows MATLAB functions to be called from COMSOL and evaluated while solving the model.

To do this, a MATLAB function was defined, whose input values are the Ca^2+^ and IP_3_ concentrations at the cluster site, as well as the time step defined in COMSOL, and the output value is a Boolean variable that defines the state of the cluster (open or closed).

The propensities are defined as follows:

${P_{C\to O}=k}_{co}\frac{N_{ca}}{\Omega}\left( \frac{N_{IP}}{N_{IP}+K_{IP}} \right)^{4}\left( 1-N_{o}-N_{i1}-N_{i2} \right)\Delta t$

$P_{O\to i1}=\frac{N_{ca}\left( N_{ca}-1 \right)\left( N_{ca}-2 \right)}{\Omega^{3}}\Delta t$

$P_{O\to i2}=k_{oi1}N_{o}\Delta t$

${P_{i1\to C}=k}_{i1c}N_{i1}\Delta t$

$P_{i2\to C}=k_{i2c}N_{i1}\Delta t$

The probability that the cluster remains in the same state is:

$P_{not}=1-P_{C\to O}-P_{O\to i1}-P_{O\to i2}-P_{i1\to C}-P_{i2\to C}$

where *k_co,_ k_oi1,_ k_oi2,_ k_i1c_* and *k_i2c_* stand for the rate constants that characterize the passages between the different states of the cluster (represented by the Boolean variables N_O_ for Open, N_C_ for Closed, N_i1_ for inhibited 1, and N_i2_ for inhibited 2), *N_Ca_* is the number of cytosolic Ca^2+^ ions and Ω is the extensivity parameter. *N_IP_* and *K_IP_* represent the number of IP_3_ molecules and the IP_3_ dissociation constant of the IP_3_R (multiplied by Ω), respectively.

The time interval $\Delta t$ (defined in COMSOL) must be small enough so that the probability that the cluster remains in the same state after $\Delta t$ is greater than 0.95. The interval [0,1] was divided into 6 sub-intervals, corresponding to the 5 possible reactions plus the case in which no reaction occurs after $\Delta t$. For this algorithm, a random number between 0 and 1 is generated. The sub-interval containing this random number then defines the state of the cluster at time $t+\Delta t$, being 1 for this and 0 for all others. The state of the cluster depends on its immediate previous state and on the IP_3_ and Ca^2+^ concentrations. It is updated at each time step.

Table S1

Propensities and evolution equations used in the simulations of puffs and spikes dynamics.

| **Event** | **Propensity functions** $\mathbf{(}\boldsymbol{\omega}_{\boldsymbol{r}}\mathbf{)}$ | **Action** |
| --- | --- | --- |
| $\boldsymbol{C}\boldsymbol{\to}\boldsymbol{O}$ | $k_{co}\frac{N_{ca}}{\Omega}\left( \frac{[{IP}_{3}]}{K_{D}+\left[ {IP}_{3} \right]} \right)^{4}\left( 1-N_{o}-N_{i1}-N_{i2} \right)$ | $N_{c}=0$  $N_{o}=1$ |
| $\boldsymbol{O}\boldsymbol{\to}\boldsymbol{I}_{\mathbf{1}}$ | $k_{oi1}N_{o}\frac{N_{ca}\left( N_{ca}-1 \right)\left( N_{ca}-2 \right)}{\Omega^{3}}$ | $N_{o}=0$  $N_{i1}=1$ |
| $\boldsymbol{O}\boldsymbol{\to}\boldsymbol{I}_{\mathbf{2}}$ | $k_{oi1}N_{o}$ | $N_{o}=0$  $N_{i2}=1$ |
| $\boldsymbol{I}_{\mathbf{1}}\boldsymbol{\to}\boldsymbol{C}$ | $k_{i1c}N_{i1}$ | $N_{i1}=0$  $N_{c}=1$ |
| $\boldsymbol{I}_{\mathbf{2}}\boldsymbol{\to}\boldsymbol{C}$ | $k_{i2c}N_{I2}$ | $N_{i2}=0$  $N_{c}=1$ |

| **Equations describing Ca^2+^ diffusion and Ca^2+^ handling mechanisms**  At a cluster site  In the cytoplasm |
| --- |
| $\frac{\partial\left[ {Ca}^{2+} \right]}{\partial t}=D_{C}\nabla^{2}\left[ {Ca}^{2+} \right]+J_{leak}-J_{SERCA}$ |
| $\frac{\partial\left[ {Ca}^{2+} \right]}{\partial t}={D_{C}\nabla}^{2}\left[ {Ca}^{2+} \right]+\text{Σ}\text{o}$ |
| $J_{SERCA}=\frac{v_{p}\left[ {Ca}^{2+} \right]^{2}}{{K_{p}}^{2}+\left[ {Ca}^{2+} \right]^{2}}$ |
| $J_{leak}=\frac{v_{p}\left[ {Ca}^{2+} \right]_{b}^{2}}{{K_{p}}^{2}+\left[ {Ca}^{2+} \right]_{b}^{2}}$ |

Table S2

Default values of the parameters used in all simulations except when mentioned explicitly.

| Parameter | Definition | Value |
| --- | --- | --- |
| k_co_ | C→O | 50 µM^-1^s^-1^ |
| k_oi1_ | O→I_1_ | 0.05 µM^-3^s^-1^ |
| k_oi2_ | O→I_2_ | 40 s^-1^ |
| k_i1c_ | I_1_→C | 0.005 s^-1^ |
| k_i2c_ | I_2_→C | 2 s^-1^ |
| ν_p_ | Maximal rate of SERCA | 0.9 µM s^-1^ |
| K_p_ | SERCA binding constant | 0.1 µM |
| [Ca^2+^]_b_ | Basal [Ca^2+^] | 0.04 µM |
| [IP_3_]_b_ | Basal [IP_3_] | 0.05 µM |
| D_C_ | Ca^2+^ diffusion coefficient | 40 µm^2^s^-1^ |
| Σ | Ca^2+^ release rate from one cluster | 500 µM s^-1^ |
| K_D_ | IP_3_ dissociation constant from IP_3_Rs | 0.1µM |
| L_c_ | Length of a compartment in the simulations | 0.5 μm |
| V_c_ | Volume of a compartment in the simulations | 10^-16^ L |
| Ω | Extensivity parameter | N_AV_ . V_c_. 10^-6^ |

1. **Incomplete Ca^2+^ buffering at 5 μM EGTA in the distributed photorelease protocol**

The average distance between clusters of IP_3_Rs in SH-SY5Y cells is ~ 2.5 μm. Over this distance, 5 μM EGTA is not sufficient to prevent communication between clusters. Indeed, the time during which an ion diffuses at random until it is captured by a buffer molecule is given by (Stern et al., 1992):

$\tau_{capture}=\frac{1}{k_{+}\left[ EGTA \right]}$ .

Given that k_+_ = 3-10 μM^-1^s^-1^ (Dargan et al., 2004), 0.02 s < $\tau_{capture}$ < 0.067s. During this time, a free Ca^2+^ ion will, on average, diffuse on a distance given by

$\lambda= \sqrt{D_{Ca}\tau_{capture}}$ .

Considering that D_Ca_ = 220 μm^2^s^-1^ that corresponds to local diffusion of free Ca^2+^ in the cytoplasm, 2.1 μm < λ < 3.8 μm. This value is approximative because it does not consider Ca^2+^ binding to endogenous immobile buffers, which is expected to be limited at the space and time scales considered.

To get a more precise idea of what is happening at the level of Ca^2+^ diffusion between clusters, one can estimate the effective Ca^2+^ diffusion coefficient that corresponds to the presence of 5 μM EGTA, i.e.


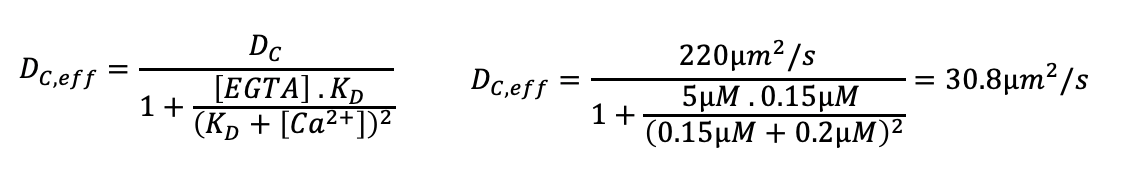


Thus, the characteristic time of Ca^2+^ diffusion on a 2.5 μm distance is of the order of 200 ms, which is in the range of the duration of Ca^2+^ increases at a puff site.

References

Dargan, S., Schwaller, B. & Parker, I. Spatiotemporal patterning of IP_3_-mediated Ca^2+^ signals in *Xenopus* oocytes by Ca^2+^ binding proteins. *J. Physiol.* **556.2**, 447-461 (2004)

Stern, M. Buffering of calcium in the vicinity of a channel pore. *Cell Calcium* **13**, 183-192 (1992)

******************


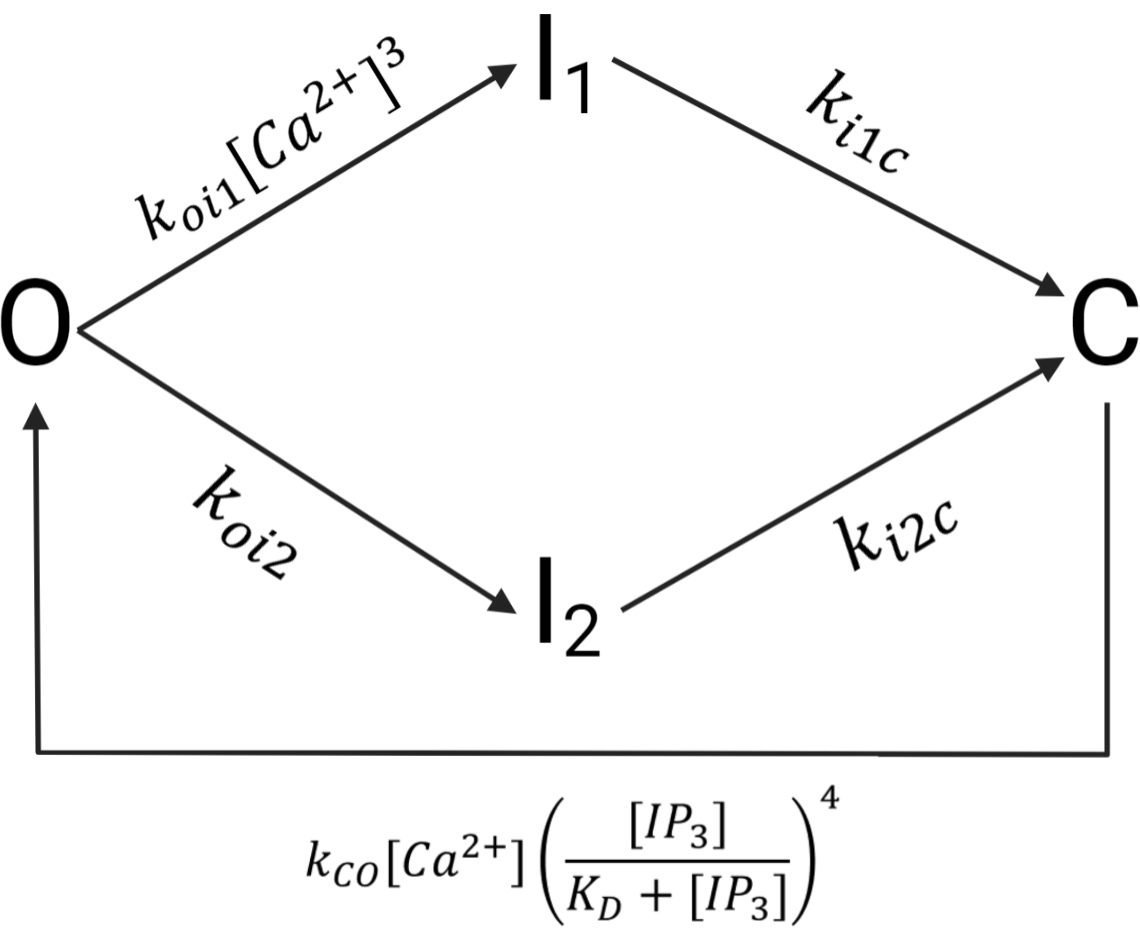


Figure S1

Schematic representation of the model used to simulate the dynamics of a cluster of IP_3_R’s. The model is taken from Calabrese et al. (2010), where it was calibrated against experimental observations. Here, we explicitly incorporate the dependence of the rate of passage of the cluster from the closed (C) to the open (O) state on [IP_3_].


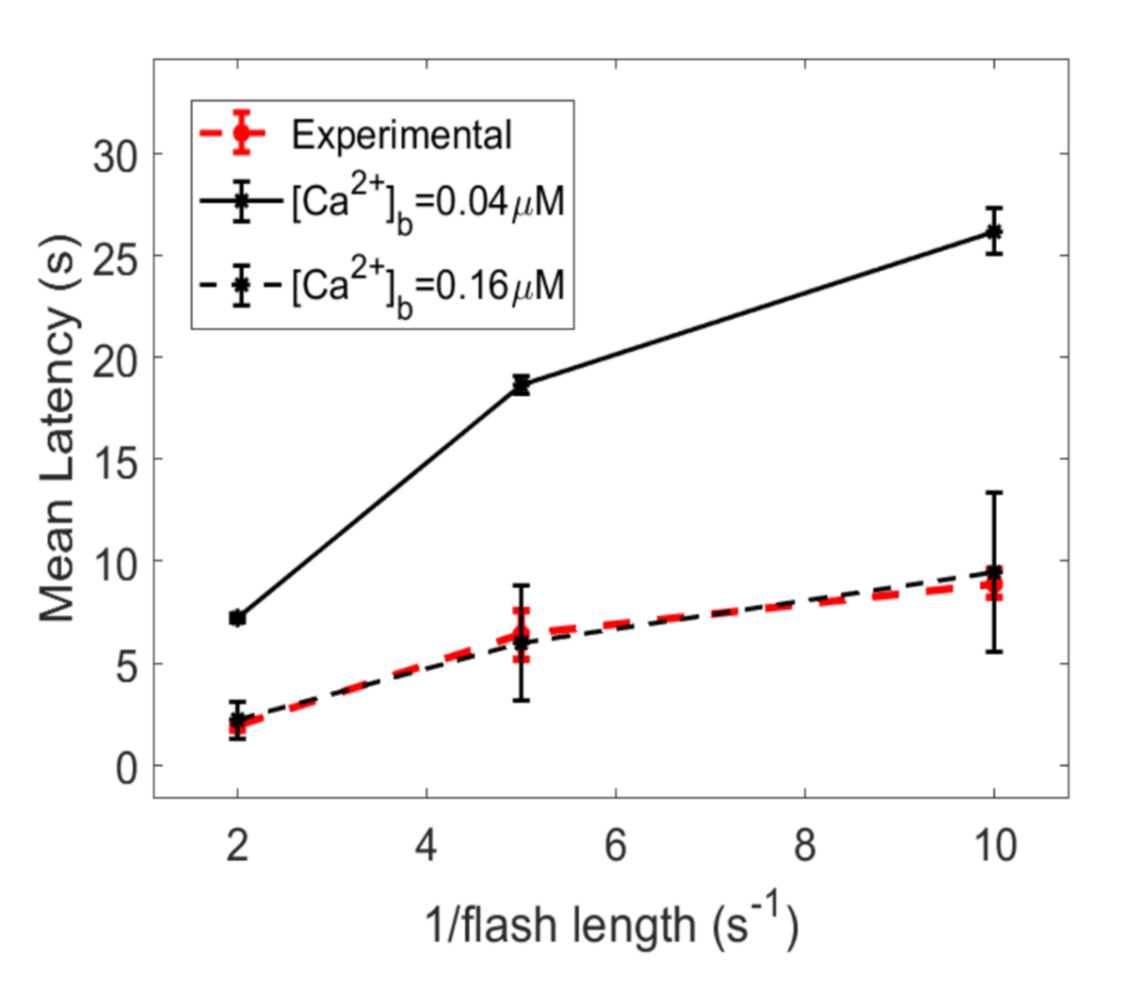


Figure S2

Mean latency as a function of the inverse of flash duration for the re-estimated value of *θ* (600 μMs^-1^) for two different values of basal Ca^2+^ concentration. The increase in basal Ca^2+^ concentration is thought to arise from the uncomplete Ca^2+^ buffering by EGTA in the experiments and corresponds to a local average Ca^2+^ increase at puff sites in the protocol of distributed photorelease. It explains why the value of *θ* directly inferred from the experiments with distributed photorelease of IP_3_ is overestimated.


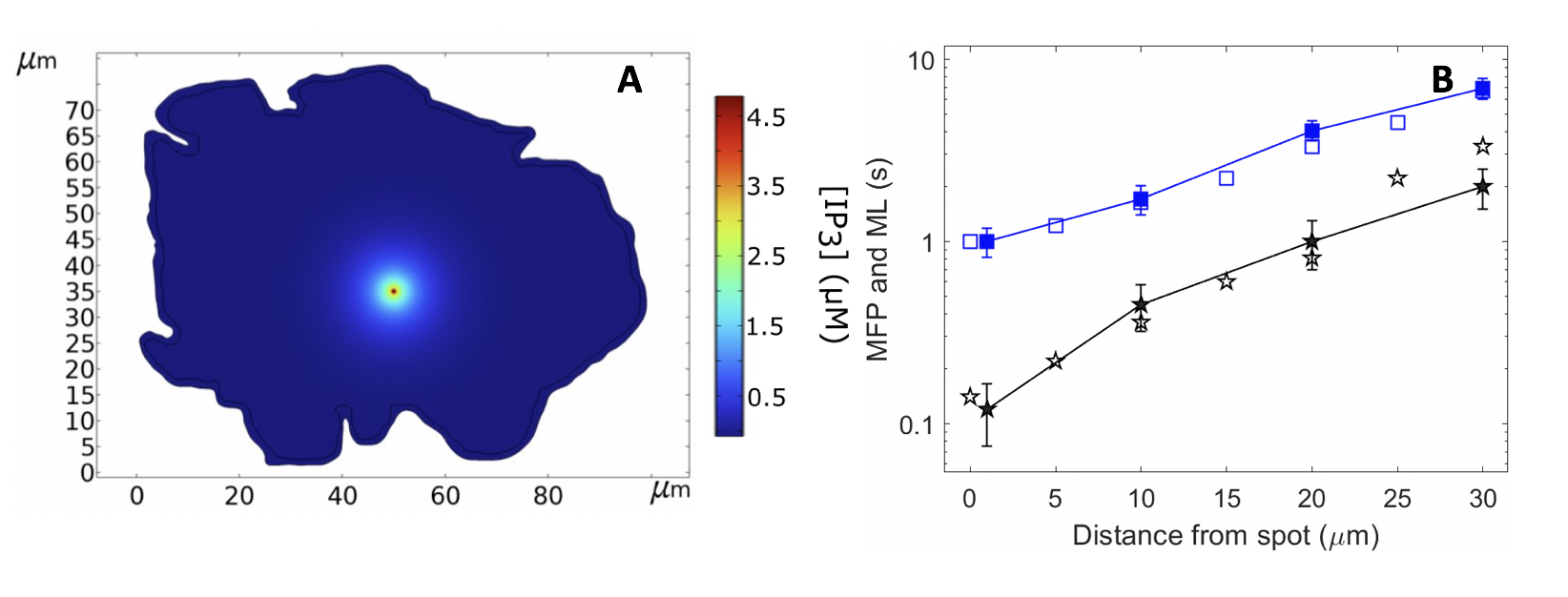


Figure S3

Simulations of Ca^2+^ puff occurrence in response to the localized photorelease of a non-metabolizable IP_3_ analog in COS-7 cells, assuming an effective diffusion coefficient of IP_3_ *D_I_* = 100 μm^2^s^-1^. Panel **A** shows the simulated cell, redrawn in COMSOL Multiphysics, with the distribution of IP_3_ concentration at the end the flash of the IP_3_ analog (θ = 3591.73 μMs^-1^). Panel **B** shows simulated (plain dots) and experimental (empty dots) mean latencies (blue) and minimal first puff latencies (black). Flash duration is 500 ms. The shape of the cell and the experimental values of latencies are taken from Dickinson et al. (2016). Simulation procedures are the same as for Figure 4 (main text).


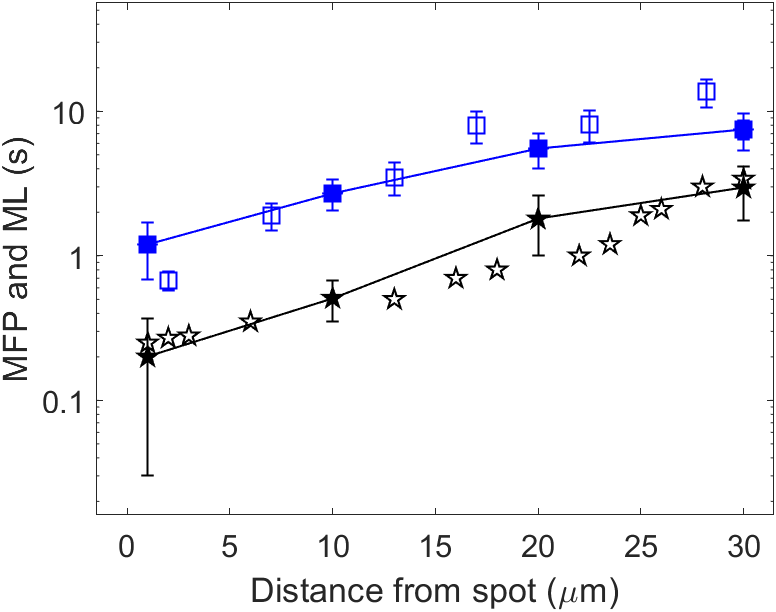


Figure S4

Simulations of Ca^2+^ puff occurrence in response to the localized photorelease of a non-metabolizable IP_3_ analog in an ellipsoidal 3D geometry, assuming an effective diffusion coefficient of IP_3_ *D_I_* = 100 μm^2^s^-1^. Results correspond to the spatio-temporal simulations shown in Figure 4. Shown are the simulated (plain symbols) and experimental (empty symbols) mean latencies (blue) and minimal first puff latencies (black). Flash duration is 500 ms. Experimental values of latencies are taken from Dickinson et al. (2016). Simulation procedures are the same as for Figure 4 (main text). The rate of localized IP_3_ photorelease, *θ* , was taken equal to 2500 μMs^-1^, which corresponds to the 250 μMs^-1^ value for the 2D case.


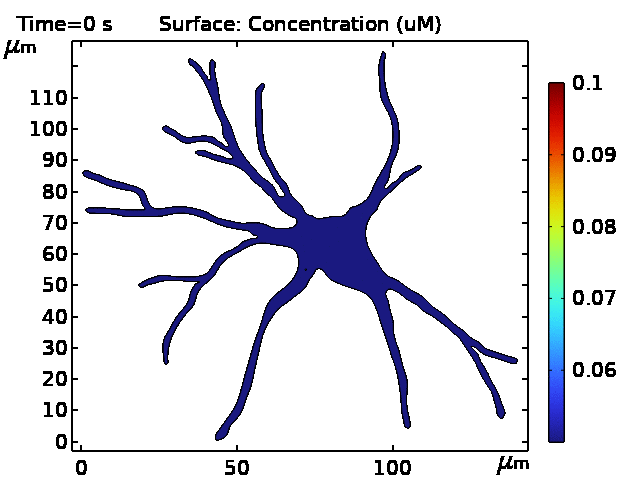


Video S5

Simulations of IP_3_ diffusion in response to the localized photorelease of a non-metabolizable IP_3_ analogue in a 2D geometry resembling an astrocyte, assuming an effective diffusion coefficient of IP_3_ *D_I_* = 100 μm^2^s^-1^. Simulation procedure is described in the legend of Figure 7 (main text).


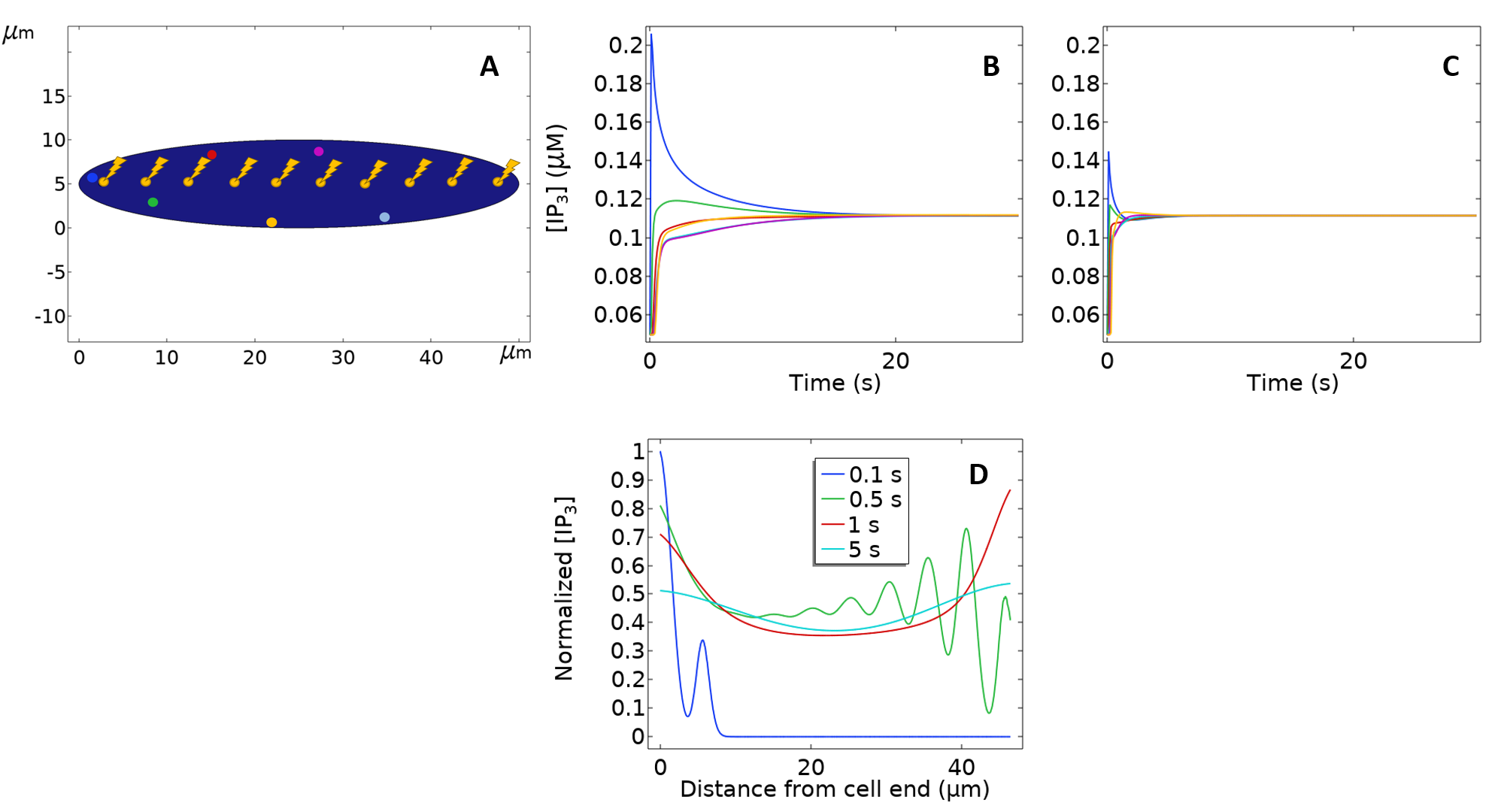


Figure S6

Simulations of the protocol of distributed photorelease used by Dickinson et al. (2016). As illustrated in panel **A**, IP_3_ is liberated at 10 different spots (1.3 μm in diameter) to provoke an increase that is supposed to be nearly homogenous in the whole cell, which is indeed indicated by the observation that puff latencies do not depend on the spatial location of the puff sites in these conditions. To reproduce the experimental protocol, IP_3_ is sequentially released during 50 ms at a rate *θ* = 250 μMs^-1^, at each spot from left to right. The total amount thus corresponds to a 500 ms flash of 250 μMs^-1^ intensity, as modelled in Figure 4. Panel **B** shows the time evolutions of the IP_3_ concentrations at the locations of the dots (clusters) of corresponding color in panel A, when *D_I_* = 10 μm^2^s^-1^. Panel **C** shows the time evolutions of the IP_3_ concentrations at the locations of the dots of corresponding color in panel A, when *D_I_* = 100 μm^2^s^-1^. Panel **D** shows normalized IP_3_ concentration along the long axis of the ellipse at different times from the beginning of the first flash in the same conditions as in panel B.
